# RNA-Seq transcriptomics and pathway analyses reveal potential regulatory genes and molecular mechanisms in high- and low-residual feed intake in Nordic dairy cattle

**DOI:** 10.1186/s12864-017-3622-9

**Published:** 2017-03-24

**Authors:** M. S. Salleh, G. Mazzoni, J. K. Höglund, D. W. Olijhoek, P. Lund, P. Løvendahl, H. N. Kadarmideen

**Affiliations:** 1 Animal Breeding, Quantitative Genetics and Systems Biology Group, Department of Veterinary and Animal Sciences, Faculty of Health and Medical Sciences, University of Copenhagen, DK-1870 Frederiksberg C, Denmark; 2 Department of Molecular Biology and Genetics - Center for Quantitative Genetics and Genomics, Aarhus University, AU Foulum, DK-8830, Tjele, Denmark; 3 Department of Animal Science - Animal Nutrition and Physiology, Aarhus University, AU Foulum, DK-8830, Tjele, Denmark; 4 Department of Bio and Health Informatics, Technical University of Denmark, DK-2800, Kgs. Lyngby, Denmark

**Keywords:** RNA-Seq, Feed efficiency, Residual feed intake, Differentially expressed genes, Pathways, Dairy cattle

## Abstract

**Background:**

The selective breeding of cattle with high-feed efficiencies (FE) is an important goal of beef and dairy cattle producers. Global gene expression patterns in relevant tissues can be used to study the functions of genes that are potentially involved in regulating FE. In the present study, high-throughput RNA sequencing data of liver biopsies from 19 dairy cows were used to identify differentially expressed genes (DEGs) between high- and low-FE groups of cows (based on Residual Feed Intake or RFI). Subsequently, a profile of the pathways connecting the DEGs to FE was generated, and a list of candidate genes and biomarkers was derived for their potential inclusion in breeding programmes to improve FE.

**Results:**

The bovine RNA-Seq gene expression data from the liver was analysed to identify DEGs and, subsequently, identify the molecular mechanisms, pathways and possible candidate biomarkers of feed efficiency. On average, 57 million reads (short reads or short mRNA sequences < ~200 bases) were sequenced, 52 million reads were mapped, and 24,616 known transcripts were quantified according to the bovine reference genome. A comparison of the high- and low-RFI groups revealed 70 and 19 significantly DEGs in Holstein and Jersey cows, respectively. The interaction analysis (high vs. low RFI x control vs. high concentrate diet) showed no interaction effects in the Holstein cows, while two genes showed interaction effects in the Jersey cows. The analyses showed that DEGs act through certain pathways to affect or regulate FE, including steroid hormone biosynthesis, retinol metabolism, starch and sucrose metabolism, ether lipid metabolism, arachidonic acid metabolism and drug metabolism cytochrome P450.

**Conclusion:**

We used RNA-Seq-based liver transcriptomic profiling of high- and low-RFI dairy cows in two breeds and identified significantly DEGs, their molecular mechanisms, their interactions with other genes and functional enrichments of different molecular pathways. The DEGs that were identified were the *CYP*’s and *GIMAP* genes for the Holstein and Jersey cows, respectively, which are related to the primary immunodeficiency pathway and play a major role in feed utilization and the metabolism of lipids, sugars and proteins.

**Electronic supplementary material:**

The online version of this article (doi:10.1186/s12864-017-3622-9) contains supplementary material, which is available to authorized users.

## Background

Feed efficiency is an important trait that should be improved to increase the sustainability and profitability of livestock production. On the one hand, there is a growing demand for food derived from dairy cattle; on the other hand, this production is associated with a high carbon footprint [1, 2], affecting the sustainability of dairy farming. Thus, there is a call for more long-term sustainable interventions. Animal genomics, particularly research regarding the potential genes that are differentially expressed in relation to an increased or a decreased efficiency of feed utilization in dairy cattle, could contribute towards achieving these goals [3]. The definition of feed efficiency in dairy animals is more complicated than that in growing animals because the catabolism of body reserves, followed by the anabolism of body reserves until the next calving period, must be considered in dairy animals [4]. The main purpose of dairy cattle is the production of milk, and it is important to select cattle that have a high efficiency in converting feed into milk. This high efficiency will lead to lower feed costs and increased profits for milk producers [5]. High feed intake and feed efficiency reflect the high production of milk (yield, fat content, protein, lactose and other milk contents) [6]. Therefore, measuring the feed efficiency is important to improve the environment and profits of milk producers.

Feed efficiency is conventionally evaluated using a conversion ratio of the feed intake to the output of the cows. Feed conversion efficiency is an expensive trait to assess and, thus, lends itself to genomic selection. Moreover, it is not sufficient to measure how much nutrients the animal uses to convert into energy to support growth, lactation and body maintenance. In the last 10 years, transcriptomics in dairy cattle has used gene expression microarrays to identify candidate genes for milk yield, protein yield, fertility and metabolic diseases, such as ketosis and milk fever [7–10]. However, only a few studies have focused on liver transcriptomic data of feed efficiency in dairy cattle, and none have focused on Nordic dairy cattle [9–11].

Residual feed intake (RFI) has been used to describe feed efficiency in animals, including beef and dairy cattle [12–15]. Residual feed intake has been defined as the difference between the actual and predicted feed intake [16]. In other words, animals with low RFI are more feed efficient compare to high RFI animals. The heritability of the RFI trait (between 0.01 and 0.38) is quite reliable as a genetic selection trait [17–19]. Hence, the RFI may be a relevant trait to consider in selecting genetically superior animals for breeding studies. Genome-wide association studies (GWAS) characterizing the gene expression and gene regulatory mechanisms related to feed efficiency are quite established in pigs (and poultry) [20, 21]; however, such studies in dairy cattle are fairly recent [22]. In this study, we used an RFI adjusted for stage of lactation, management group, breed and parity. Given the major role of the liver in regulating nutrient homeostasis [23], it is important to understand the biological mechanisms underlying this process. Thus, genome-wide gene expression studies of the liver can provide biological insights into feed processing efficiency and help to determine the mechanism(s) of feed efficiency.

Transcriptomic analyses are useful for studying animal production and health [24] and have become important components of systems genomic or systems biology methods [25]. Transcriptomic analyses provide a snapshot of all the gene expression profiles in a given tissue and insight into the gene functions pertaining to a particular trait [24]. Microarray technologies have been the main platform for animal science research in recent years; however, this trend has been increasingly replaced by RNA-Seq technologies [24–26].

The primary objective of the present study was to identify potential regulatory genes and molecular pathways involved in RFI in dairy cattle by characterizing the liver transcriptome based on RNA-Seq technologies [24, 26]. Another objective of this study was to evaluate the effects of different diets interacting with high- and low-RFI cattle and the resulting impact on the gene expression profiles and associated pathways. This study reports important findings regarding potential regulatory genes and the pathways underlying feed efficiency in dairy cattle using next-generation sequencing or RNA-Seq technology and, most importantly, the nutrigenomics aspects of RFI x Diet interactions.

## Results

### Mapping statistics summary

The sequencing generated, on average, 57,149,474 raw reads (28,574,737 paired reads) per sample. On average, 91% of the read pairs (26,067,856 read pairs) uniquely mapped to the bovine reference genome from the Ensembl database, release 82. On average, 62% of the read pairs mapped to exonic regions, 20% of the read pairs mapped to intronic regions and almost 18% of the read pairs mapped to intergenic regions (Table 1). After quantifying the expression of the 24,616 genes annotated from the *Bos taurus* reference genome, we excluded a total of 12,591 and 12,711 genes from the remainder of the analyses (because of low expression) of the Holstein and Jersey datasets, respectively. In total, 12,025 genes in the Holstein breed and 11,905 genes in the Jersey breed were used for the subsequent analyses.

**Table 1:** Summary of the average statistics of the sequence quality and alignment information for the Jersey and Holstein breeds

	Jersey	Holstein
Number of input read pairs	29,428,257	28,221,217
Uniquely mapped read pairs	26,386,656	25,749,055
Mapping rate (%)	91.25	91.24
Exonic	62.15	62.08
Intronic	20.19	20.07
Intergenic	17.66	17.86

### Differentially expressed genes (DEGs)

**Fig. 1:**
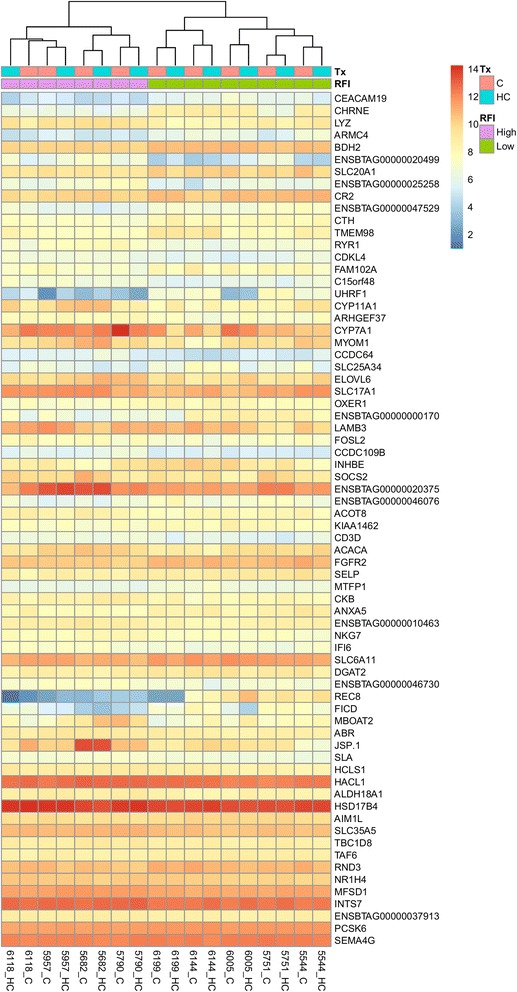
Heatmap showing the gene expression data of the 70 significantly differentially expressed genes (padj < 0.05) annotated with the gene ID in Holsteins without the interaction term. The data are log2 normalized. Tx = Treatment diet; C = Control diet; HC = High concentrate diet; RFI = Residual Feed Intake groups

**Fig. 2:**
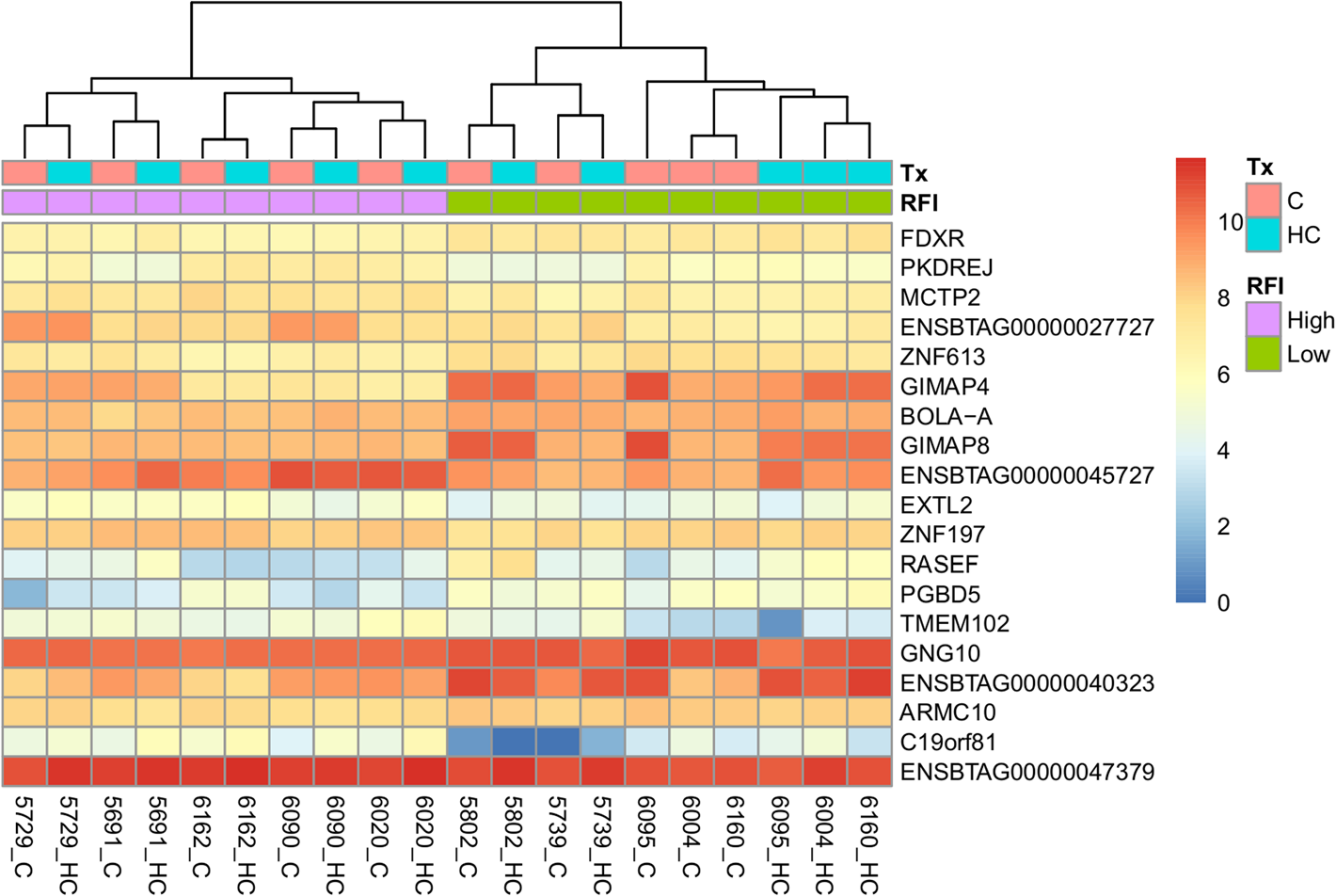
Heatmap showing the gene expression output of the 19 significantly differentially expressed genes (padj < 0.05) annotated with the gene ID in Jerseys without the interaction term. The data are log2 normalized. Tx = Treatment diet; C = Control diet, HC = High concentrate diet; RFI = Residual Feed Intake groups

The DEGs identified by DESeq2 are shown in the heat map (Figs. 1 and 2).

**Fig. 3:**
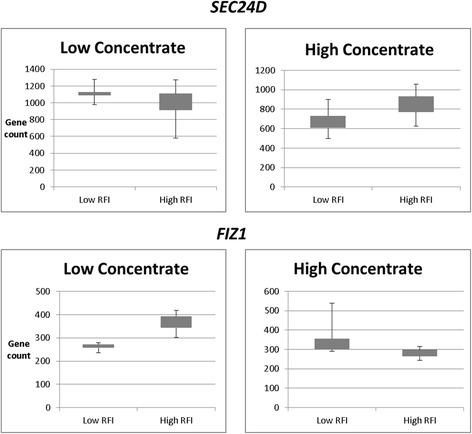
The plot counts of the 2 genes that show a significant change (padj < 0.05) greater than 0.5-fold in the interaction analysis in the Jerseys

The interaction analysis showed low numbers of DEGs in both diet groups (Table 2). From the DESeq2 output, 22 genes and 14 genes in the Holstein and Jersey breeds, respectively, were detected as significant DEGs for the interaction between RFI and diet. No significantly DEGs were identified for the interaction in the Holstein group. However, in the Jersey group, two genes, SEC24 Homologue D (*SEC24D*) and FLT3-Interacting Zinc Finger 1 (*FIZ1*), were differentially expressed in the FE groups, depending on the two diet types (Fig. 3).

**Table 2:** Number of differentially expressed genes between high- and low-RFI in a separate diet group in the model with an interaction term and without an interaction term (the diet group was pooled together) according to corrected *p*-values < 0.05

	Control	High concentrate	With interaction	Without interaction
Holstein	9	13	0	70
Jersey	6	6	2	19

Furthermore, 70 Holstein and 19 Jersey DEGs were identified by comparing the RFI status directly without accounting for an interaction (Table 2) (Figs. 1 and 2). Nine genes in the Holstein breed and five genes in the Jersey breed were not annotated. The list of DEGs with their fold changes in the Holstein and Jersey cows is shown in Additional files 1 and 2.

### Overrepresented pathways and gene networks

The GOseq analysis did not identify any significantly enriched GO (Gene Ontology) terms or KEGG (Kyoto Encyclopedia Genes and Genomes) pathways.

The output of the GSEA (Gene Set Enrichment Analysis) is presented in Tables 3, 4 and 5, which show the most significantly enriched pathways with FDR (False Discovery Rate) *q*-values less than 0.01. We identified seven overrepresented pathways for the downregulated set of genes, and none were identified for the upregulated genes in the Holsteins. In the Jerseys, two pathways were overrepresented for genes with negative-fold changes, and three pathways were overrepresented for genes with positive-fold changes. The top KEGG pathways for the genes that were downregulated in the high-RFI group in the Holsteins and the Jerseys is the primary immunodeficiency pathway, while the significant pathways identified for the genes that were upregulated in the high-RFI group were only detected in the Jerseys. We also identified that most of the pathways within the strong indication thresholds (FDR *q*-value <0.05) were related to the metabolism of retinols, starch, sucrose, ether lipids and drugs.

**Table 3:** KEGG pathways identified for the downregulated genes in the high-RFI group with an FDR *q*-value < 0.01 from the output of the GSEA in the Holsteins

	Name	FDR *q*-value	Core enrichment gene
1	Primary immunodeficiency	~0	*CD3D, IL7R, PTPRC, JAK3, ZAP70, CD3E, LCK, ADA, CD8A, BTK, TAP1, UNG, RFX5, CD4*
2	Natural killer cell mediated cytotoxicity	~0	*NFATC2, TNFRSF10D, NCR3, ICAM1, RAC2, ZAP70, PIK3CG, GRB2, LCK, NFAT5, PTK2B, LCP2, PRF1, ITGAL, TYROBP, PIK3CD, SH2D1A, TNF, VAV1, TNFSF10, PLCG2, ITGB2, PAK1, PIK3R5, KRAS, PRKCA, FASLG, SYK, LAT, CD48, IFNGR1, PIK3CA, FCER1G, KLRK1, RAF1, PTPN11, FAS, IFNAR1, PTPN6, HRAS, SOS2, PRKCB*
3	T cell receptor signaling pathway	~0	*CD3D, NFATC2, JUN, PTPRC, ITK, CD3G, ZAP70, CD3E, PRKCQ, PIK3CG, GRB2, LCK, NFAT5, CD8A, RASGRP1, LCP2, TEC, CARD11, PIK3CD, TNF, VAV1, NFKBIA, PAK1, PIK3R5, KRAS, MAPK9, CD4, PDK1*
4	Leukocyte transendothelial migration	0.002	*GNAI1, NCF1, RAPGEF4, OCLN, ITK, CLDN2, ICAM1, RAC2, CLDN1, NCF4, PIK3CG, CDH5, CXCL12, EZR, PTK2B, ITGAM, CLDN4, CYBB, ITGAL, NCF2, PIK3CD, VAV1, CLDN15, PLCG2, ITGB2, PIK3R5, PRKCA, MYL12B, ARHGAP35, F11R, ROCK2, RAP1A, ITGB1, ITGA4, PIK3CA, CXCR4, MSN, CTNNB1*
5	Chemokine signaling pathway	0.002	*CXCR6, GNAI1, CCR2, NCF1, ITK, CXCL9, DOCK2, CCL14, PLCB2, RAC2, JAK3, HCK, PIK3CG, GRB2, CX3CR1, CXCL12, ADCY7, ELMO1, PTK2B, GRK5, CCR5, WAS, ARRB1, PIK3CD, ADRBK2, VAV1, LYN, NFKBIA, PAK1, PIK3R5, KRAS, GNB4, GNG2, PRKX, FGR, STAT3, ROCK2, GNB5, RAP1A, PLCB1, STAT1, IKBKG, AKT3, CHUK, PIK3CA, CXCR4, GNG10, PRKACB*
6	FC gamma R mediated phagocytosis	0.008	*NCF1, SCIN, PTPRC, DOCK2, RAC2, HCK, ARPC1B, MYO10, PIK3CG, MARCKS, LIMK1, PLA2G4A, WAS, INPP5D, PIK3CD, ASAP1, VAV1, PLCG2, LYN, PAK1, PIK3R5, PRKCA, SYK, ARPC1A, PIKFYVE, LAT, PLD2, ARPC3, AKT3, PIK3CA*
7	Propanoate metabolism	0.009	*ACACA, ACSS2, ACAT2, ACACB, EHHADH*

FDR *q*-value = adjusted *p*-value; core enrichment gene = subset of genes that contributes most to the enrichment result

**Table 4:** KEGG pathways identified for the downregulated genes in the high-RFI group with an FDR *q*-value < 0.01 from the output of the GSEA in the Jerseys

	Pathways name	FDR *q*-value	Core enrichment gene
1	Leukocyte transendothelial migration	0.006	*NCF1, PLCG1, ICAM1, VAV1, MAPK12, MSN, PTK2, SIPA1, ITGAM, NCF4, RAP1B, VASP, PIK3CD, RHOH, PIK3R5, RAC2, RAPGEF3, ITK* *CLDN14, THY1, MYL12B, CLDN4, CXCR4, ACTG1, ITGB2, CYBA, CLDN7, EZR, CYBB, CLDN1, GNAI1, NCF2, MMP2, PRKCB*
2	Primary immunodeficiency	0.010	*CD4, CD8A, ADA, PTPRC, JAK3, TAP1, ZAP70, CD3e, CD3D, LCK*

FDR *q*-value = adjusted *p*-value; core enrichment gene = subset of genes that contributes most to the enrichment result

**Table 5:** KEGG pathways identified for the upregulated genes in the high-RFI group with an FDR *q*-value < 0.01 from the output of the GSEA in the Jerseys

	Pathways name	FDR *q*-value	Core enrichment gene
1	Retinol metabolism	0.002	*PNPLA4, CYP2B6, CYP2C18, RETSAT, CYP1A1, RDH11, CYP1A2, ALDH1A1, CYP26B1, LRAT, ADH5, UGT2A3, RDH16, UGT1A1, ALDH1A2, RDH10*
2	Metabolism of xenobiotics by cytochrome P450	0.003	*CYP2B6, ALDH1A3, CYP2E1, CYP2C18, EPHX1, CYP1A1, CYP1A2, MGST1, MGST3, ALDH3B1, ADH5, UGT2A3, UGT1A1*
3	Ether lipid metabolism	0.009	*ENPP6, PLA2G7, PLD2ENPP2, LPCAT2PLA2G12A, AGPSPLD1*

FDR *q*-value = adjusted *p*-value; core enrichment gene = subset of genes that contributes most to the enrichment result

**Fig. 4:**
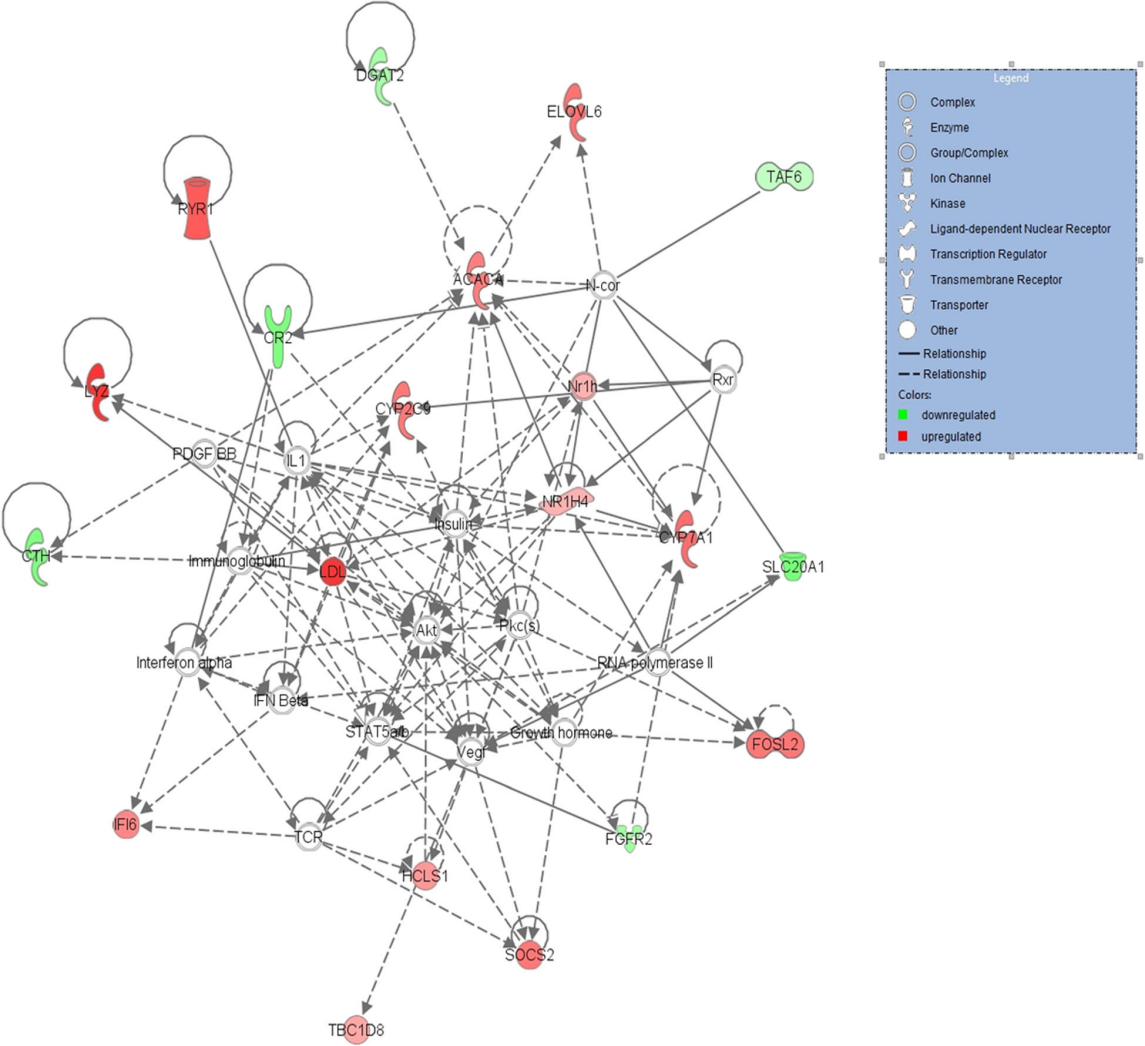
The relationship between 18 DEGs in network 1 in the Holsteins. The genes highlighted in red were upregulated, while those highlighted in green were downregulated in the high-RFI group

The networks identified from the DEGs by IPA® (Ingenuity® Pathway Analysis) are presented in Tables 6 and 7. Seven and six networks were identified for the Holsteins and Jerseys, respectively. The top networks (Fig. 4) in the Holsteins involved 18 genes that are implicated in metabolic diseases, endocrine system disorders and gastrointestinal diseases. The genes that were upregulated in the high-RFI group in the top network of the Holsteins were *ACACA, CYP2C9, CYP7A1, ELOVL6, FOSL2, HCLS1, IFI6, NR1H4, RYR1, SOCS2* and *TBC1D8*; while the downregulated genes were *CR2, CTH, DGAT2, FGFR2, SLC20A1* and *TAF6*.

**Table 6:**
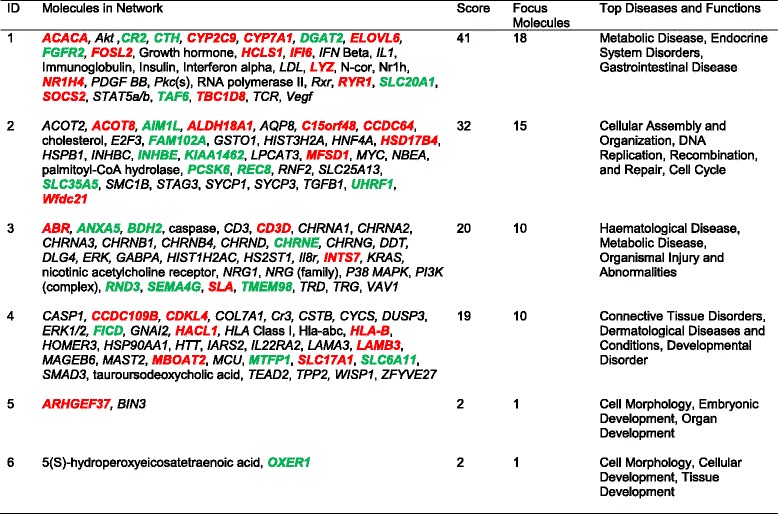
Gene networks from the 58 differentially expressed genes for the Holstein group converted to human orthologous genes

**Table 7:**
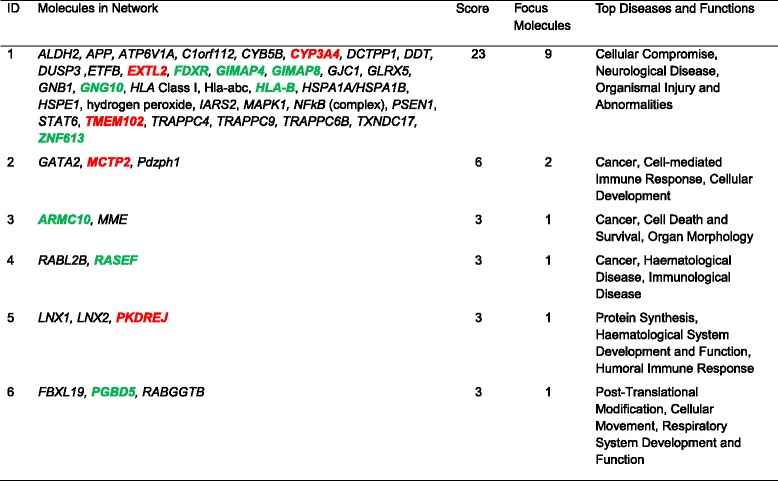
Gene networks from the 15 differentially expressed genes for the Jersey group converted to human orthologous genes

**Fig. 5:**
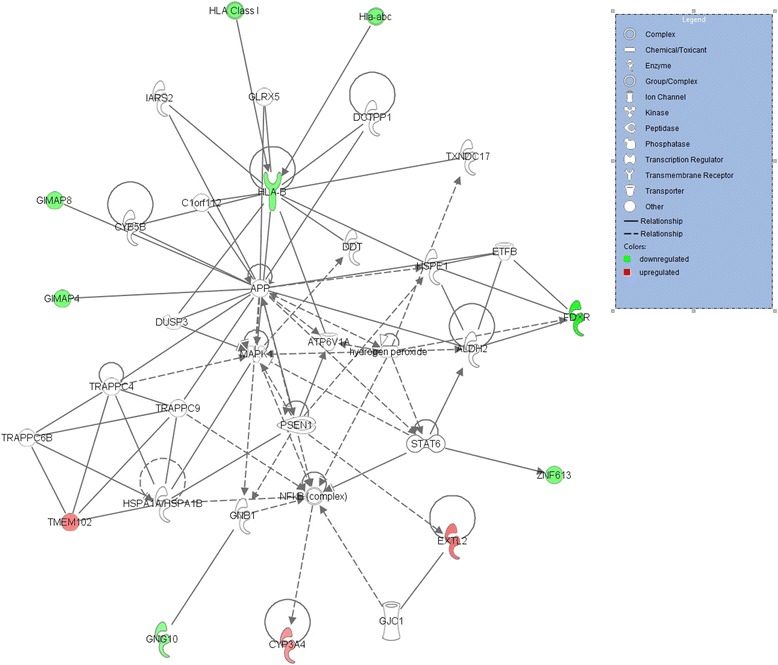
The relationship between the nine DEGs in network 1 in the Jerseys. The genes highlighted in red were upregulated, while those highlighted in green were downregulated in the high-RFI group

The top networks in the Jerseys (Fig. 5) involve nine genes that are implicated in cellular compromise, neurological disease, organismal injury and abnormalities. The network includes the genes *CYP3A4, EXTL2* and *TMEM102,* which were upregulated in the high-RFI group, and the genes *FDXR, GIMAP4, GIMAP8, GNG10, HLA-*B and *ZNF613,* which were downregulated in the high-RFI group.

To investigate the DEGs interacting with each other, we analysed the candidate DEGs using the STRING 10 database. Several interacting genes were identified in the Holsteins. In particular, *ACACA* interacts with *BDH2, DGAT2, CYP11A1, HSD17B4, ALDH18A1, HACL1 and ELOVL6*. In the Jerseys, only *GIMAP4* and GIMAP8 interact with each other. The top DEGs present in the IPA network are discussed.

## Discussion

### Differentially expressed genes

The liver plays an important role in regulating the nutrient supply [27]. Hence, the liver transcriptome may lead to the identification of genes that are important for regulating feed efficiency [28, 29]. Understanding the mechanisms of action and biological functions of the highly significant DEGs in high- versus low-RFI animals experimentally tested under controlled versus high concentrate diets improves our understanding of the biology of feed efficiency in dairy cattle.

The results of this study show a robust relationship and interaction between certain genes involved in feed utilization, partitioning of energy and metabolism. The potential regulatory genes that show a positive effect on RFI were reported in this study.

Almost all the DEGs in the interaction analysis were also present in the analysis without the interaction term. This result may be due to the treatment diet (either low or high concentrate), which might not have a significant impact or be reflected in the differences in the gene expression in the Holsteins. A similar effect was observed in the Jerseys. However, we obtained a smaller number of DEGs compared to those in the Holsteins, which could be due to the small variation among the individuals in the Jersey high and low RFI groups. However, it should be noted that the number of animals from each breed is rather small and could have biased the results.

Significantly enriched GO terms and pathways were not identified by GOseq; therefore, we focused on a number of genes that appeared several times in significant networks in the IPA, GSEA and STRING 10. Hence, *ACACA* (Acetyl-CoA Carboxylase Alpha), *CYP11A1* (Cytochrome P450, Family 11, Subfamily A, Polypeptide 1), *CYP2C9* (Cytochrome P450, Family 2, Subfamily C, Polypeptide 9) *BDH2* (3-Hydroxybutyrate Dehydrogenase, Type 2), *DGAT2* (Diacylglycerol O-Acyltransferase 2), and *FBP2* (Fructose-1,6-Bisphosphatase 2) in the Holsteins and *CYP3A4* (Cytochrome P450, Family 3, Subfamily A, Polypeptide 4) and *FDXR* (Ferredoxin Reductase) in the Jerseys were chosen to gain a better understanding of the role of the top genes and networks that were involved. Some of the DEGs reported in previous reports [10, 28, 30] were found to be involved in similar processes related to feed utilization in humans, ruminants and other mammals.

Recently, an investigation of two divergent RFI groups in beef cattle using RNA-Sequencing [28, 31] revealed eight and seven significantly DEGs, respectively. However, similar DEGs were not identified in the present study on dairy cattle, suggesting that the discrepancy may be based on the breed. However, some of our results are consistent with a study showing a connection between immune function and most of the DEGs associated with low and high RFI in beef cattle [31]. Alexandre et al. (2015) [28] concluded that the DEGs related to feed efficiency and hepatic physiology were focused more towards the immune response, the metabolism of lipids and cholesterol and hepatic inflammation, which is also consistent with the findings of the present study.

### Insights from the Gene Set Enrichment Analysis (GSEA)

Primary immunodeficiency was the top overrepresented pathway detected by the GSEA. This pathway is present and significantly enriched in both cattle breeds. It was stated in the details of the pathway that primary immunodeficiency is a heterogeneous group of disorders. The downregulation of the primary immunodeficiency pathway in the high-RFI cows in both breeds suggests that a low immunity may affect the efficiency of feed utilization. Ozuna et al. (2012) [32] observed that primary immunodeficiency disorder is consistently inherited by low-feed efficiency pigs. Consistently, Kogelman et al. (2014) [33] and Do et al. (2013) [34] reported a correlation between genes related to immunodeficiency function disorders or immunity-related diseases and low-feed efficiency in pigs.

The results of the enrichment and pathway analysis of the DEGs contributes towards the understanding of the function of these genes in relation to the efficiency of feed utilization. The steroid hormone biosynthesis pathway was one of the top KEGG pathways identified in an analysis of negative energy balance in dairy cows [10]. We also discovered that this pathway was overrepresented in the set of genes that were upregulated in the high-RFI group in the Jersey cows (FDR *q*-value < 0.05). Steroid hormone biosynthesis should always occur in the adrenal glands and gonads, while the liver is the site of steroid hormone inactivation. The upregulation of this pathway indicated that steroid hormones were inactivated in the high-RFI group. Therefore, we could conclude that this pathway plays an important role in FE. Furthermore, both *CYP11A1* and *CYP7A1,* which function in cholesterol homeostasis, were identified as DEGs in our experiments, and they are a part of this KEGG pathway.

Additional interesting KEGG pathways that were upregulated in the high-RFI Jersey group were involved in xenobiotics metabolism, retinol metabolism, sphingolipid metabolism, starch and sucrose metabolism, ether lipid metabolism, arachidonic acid metabolism and drug metabolism cytochrome P450. Most of these pathways (Additional file 3) were related to nutrients (fatty acids, carbohydrates and proteins) and metabolism. de Almeida Santana et al. (2016) [35] reported that the retinol metabolic pathway was involved in the feed conversion ratio in beef cattle in relation to rump fat thickness. The authors also discussed that lipid and protein metabolisms were well-known important factors in feed efficiency physiology. The relationship between retinol metabolism and the feed conversion ratio phenotype in Nellore beef cattle has been previously described [36] and [35].

The top pathway of the metabolism of xenobiotics by cytochrome P450 involved the *CYP* genes. Specifically, the *CYP11A1* gene was upregulated in the high-RFI group compared with that in the low-RFI group. The *CYP11A1* gene was not present in the IPA output because it has no Entrez gene ID when uploaded as an input. However, *CYP11A1* was also identified as a DEG in the Holstein group. The *CYP11A1* gene is also known as cytochrome P450, which functions in drug metabolism and cholesterol, steroid and lipid synthesis. When the expression of this gene is high, it will also lead to the active synthesis of lipids, steroids and hormones. Yi et al. (2015) [29] have mentioned that the upregulation of *RSAD,* which is a gene that has a similar function to *CYP11A1* in the low RFI (high feed efficiency) group, may lead to a decreased feed intake, high energy utilization and few energy costs by modulating fatty acid and leptin metabolism. These results are consistent with those reported by McCabe et al. (2012) [10], who discovered that *CYP11A1* was upregulated in severe negative energy balanced cows. This result suggests that the *CYP11A1* gene indeed played an important role in lipid synthesis and the regulation of cholesterol synthesis in the liver. Together with *CYP11A1*, the *CYP7A1* and *CYP2C9* genes were also differentially expressed and had the same pattern of expression in the Holsteins. In another study conducted by [37], the *CYP* genes were involved in steroidogenesis and converted cholesterol into pregnenolone and then to dehydroepiandrosterone (DHEA). The *CYP* gene function was also discussed in feed efficiency, particularly pertaining to hepatic metabolism [28, 38, 39].

### Ingenuity® Pathways Analysis (IPA®) output and interactions between DEGs

The output of the IPA for the Holsteins showed the top networks of the 18 upregulated DEGs, which included Metabolic Diseases, Endocrine System Disorders and Gastrointestinal Diseases. Consistently, the network of metabolic diseases was associated with the differential gene expression in the severe negative energy balance in high-yielding cows [11]. The metabolic disease network may be closely related to the immune system. Paradis et al. (2015) [31] have stated that immunity is very important to produce animals that have less energy to fight against systemic inflammation, have better detoxification of endotoxins and use more energy for growth.

The output from the STRING 10 analysis shows that among the significantly DEGs, the *ACACA* gene has interactions with *CYP11A1, BDH2, DGAT2, HSD17B4, FGFR2, HACL1* and *ALDH18A1*. This interaction depicts the importance of the *ACACA* gene in this output. The function of the *ACACA* gene is to convert acetyl CoA to fatty acids, also known as lipogenesis. The upregulation of the *ACACA* gene in the high-RFI Holstein group in this network is also interesting in relation to functions in feed utilization. A positive relationship has been reported between ACACA enzyme activity and intramuscular fat levels [40]. In addition, the negative relationship between the *ACACA* gene and other lipogenesis pathway genes and milk production in dairy cattle was also confirmed by Sumner-Thomson et al. (2011) [41]. Hence, the increased *ACACA* gene expression might reflect the deposition of fat in the high-RFI cows.

The output of the DEG analysis revealed that *BDH2* is another interesting gene to be considered due to its downregulation in the high-RFI cattle. These genes play an important role in metabolism and synthesis and are very well known for their role in the degradation of ketone bodies. In contrast, no change was observed in the transcript abundance of genes involved in ketone body synthesis [3-hydroxy-3-methylglutaryl-CoA synthase 2 (*HMGCS2*), 3-hydroxybutyrate dehydrogenase, type 2 (*BDH2*)] in cows subjected to nutrient restrictions to reduce the frequency of milking [42]. However, our results showed a downregulation of *BDH2* genes in the high-RFI cattle, suggesting that this group was inefficient in degrading ketone bodies.

In the present study, an upregulation of the *DGAT2* gene was observed in the high-RFI Holsteins. In humans, the *DGAT2* gene was reported to be a candidate for the dissociation between fatty liver and insulin resistance [30], and this result has also been observed in mice [43]. The *DGAT* gene functions in the liver by catalysing the final reaction in the synthesis of triglycerides in which diacylglycerol is covalently bound to long chain fatty acyl-CoAs. The *DGAT* gene might be a candidate for treating obesity in humans because the increased expression of *DGAT* led to obesity in mice that were resistant to diet-induced obesity [44].

The IPA analysis output for the Jersey breed showed the top overrepresented networks, involving nine DEGs that are related to cellular compromises, neurological disease, organismal injury and abnormalities. These processes appear to be closely related to the top primary immunodeficiency output from the GSEA KEGG pathways. The importance of this related pathway was previously explained as it pertains to Holsteins. The output from STRING 10 showed only one interaction between the *GIMAP4* and *GIMAP8* genes among the significantly DEGs in the Jerseys. Although the Jersey DEGs differed from those in the Holsteins, some genes have similar functions, such as the *CYP3A4* gene.

The ferredoxin reductase (*FDXR*) gene was also a top DEG in the Jersey breed. The *FDXR* gene encodes a 50,000 kDa mitochondrial flavoprotein attached to the matrix side of the inner mitochondrial membrane. FDXR transports electrons from NADPH via the soluble single electron shuttle ferredoxin to a membrane-integrated cytochrome P450 enzyme (*CYP11A1*). The upregulation of the *FDXR* gene, which occurs in the low-RFI Jersey group, can deplete the levels of the reduced NADPH. This *FDXR* gene is also known to be involved in cholesterol metabolism, which is also a part of steroid metabolism.

In addition, it is interesting to note that the *GIMAP4* and *GIMAP8* genes, which were upregulated in the low-RFI Jersey group, are also related to an immuno-associated nucleotide (IAN) subfamily of nucleotide-binding proteins. This is important for controlling the immune system and responding to infections [45]. These genes have never been implicated or previously described in relation to feed efficiency or utilization in any species. The expression consistency of these two genes is interesting to relate to the biological functions that are important for controlling the immune system. Consistent with the GSEA output results, primary immunodeficiency is the top pathway and is reflected by the differential expression of these two related genes. The *GIMAP4* and *GIMAP8* genes require further investigation regarding their importance in controlling the immune system.

### Genes in the RFI x Diet interaction in the Jersey cattle

The DEGs involved in the interaction between RFI and diet were also associated with immunodeficiency, which was a key pathway consistently identified in this study. It is interesting that the diet has an impact on genes belonging to the immunodeficiency pathway, and this result paves the way for future studies to determine how to improve diet in relation to the genetic background of the animals. Two protein-coding genes, *SEC24D* and *FIZ1,* were differentially expressed in response to the diet and were associated with pathways, including Immune System and Transport to the Golgi and subsequent modification and were involved in transcriptional regulation [45]. These genes might also be factors in the primary immunodeficiency pathway that was detected as significantly overrepresented in this study. The lack of a more extensive differential gene expression response indicates that the differences in the concentrate composition of the diet tested in this analysis may not have been sufficient to influence gene expression levels.

### Implications for improving feed efficiency via breeding

Through the integration of the information obtained from the DEGs, functional enrichment, pathway analysis and published data, this study provides a list of candidate genes whose functions and expression levels are strongly related to RFI. These candidate genes can be used to develop genomic biomarkers, eQTLs (expression quantitative trait loci), CNV (Copy Number Variation), SNPs (single-nucleotide polymorphisms) and additional markers for possible inclusion in genomic selection methods utilizing functional information [e.g., sgBLUP (system genomic BLUP) [24] and BLUP|GA (BLUP approach given the Genetic Architecture] [26].

This study was conducted with relatively small sample sizes (10 samples in each breed) but in a highly controlled environment. However, it is recommended that this study should be replicated with a larger sample size for the eventual validation of our findings.

## Conclusion

This study investigated the liver transcriptome of high- versus low-RFI animals experimentally tested with control versus high concentrate diets. The results provide an important understanding of the biology of feed efficiency in dairy cattle and a basis for elucidating the mechanisms of action and biological functions of highly differentially expressed genes. This study is novel in at least two aspects as follows: one in terms of the species/breed (dairy cattle: Danish Holsteins and Danish Jerseys) and the second in terms of the RFI x Diet experiments. Furthermore, to the best of our knowledge, this study is the first study conducted exploring residual feed intake in Nordic dairy cattle using RNA-Seq, which is known as the most accurate technology for genome-wide gene expression studies. The results reveal differences in the biological mechanisms related to residual feed intake in the Holsteins and Jerseys. The study identified 70 and 19 candidate genes that are involved in the regulation of feed efficiency pathways in the Holstein and Jersey cattle, respectively. The candidate genes identified in this study will be useful for explaining the biological effects of genomic markers in genomic selection methods utilizing functional information.

## Methods

### Animal ethics statement

In this study, individual cows of the two main dairy cattle breeds in Denmark, Holstein and Jersey, were obtained from Danish Cattle Research Centre (DCRC), Aarhus University, Denmark. The data from this herd have previously been used in quantitative genetic studies regarding feed or dry matter intake [46]. The experimental animal procedures were approved by the Danish Animal Experimentation Inspectorate.

### Animals experiments

Ten Jersey and ten Holstein cows were selected from a research herd of 200 animals. However, one of the Holstein cows was excluded from the study due to an unsuccessful liver biopsy. Animals of both breeds were divided into the following two groups: high- or low-residual feed intake (RFI). Residual Feed Intake was defined using the one-step approach [14]. Here, the random animal solutions were extracted from a random regression model in which the dry matter intake was regressed to the following fixed effects: weeks of lactation, the management group in which the cows were held, and the interaction between weeks of lactation, breed and parity. Fixed linear regressions were applied to adjust for the metabolic body weight, daily live weight change, daily body condition score change (fitted with a Legendre polynomial), and energy corrected milk yield. The random effects were cow within the breed and cow within the breed and parity. Cows were ranked based on their random effect solutions. From the available cows, blocks were defined to include two Holstein and two Jersey cows in a similar lactation stage and a similar parity group (first or older), and included one high and one low ranked cow of each breed. In total, five blocks were defined, and the cows within blocks were then allocated to the experimental treatments and measurements*.*

Table 8 shows the RFI values of the individual cows that were used for the samples and analysis. Table 8 also shows the assignment of the treatments for the first and the second periods of the experiment.

**Table 8:** Details of the experimental cows. The cows have been classified according to the breed, parity, block, RFI value, RFI group and the allocation of the diet for the first and second period. RFI values refer to the random animal solutions as explained in the text

Cow ID	Breed	Parity	Block	RFI value	RFI group	1st period	2nd period
6199	Holstein	1	5	-0.395	High	HC	C
5751	Holstein	3	2	-0.622	High	HC	C
6118	Holstein	1	3	-0.03	Low	HC	C
5957	Holstein	2	1	0.885	Low	HC	C
5790	Holstein	2	4	0.101	Low	HC	C
6004	Jersey	2	1	-1.705	High	HC	C
5739	Jersey	3	4	-0.042	High	HC	C
6090	Jersey	1	5	0.493	Low	HC	C
6162	Jersey	1	3	0.803	Low	HC	C
5729	Jersey	3	2	0.938	Low	HC	C
6144	Holstein	1	3	-1.103	High	C	HC
6005	Holstein	2	1	-1.046	High	C	HC
5544	Holstein	3	4	0.05	High	C	HC
5682	Holstein	3	2	0.695	Low	C	HC
6160	Jersey	1	5	-0.511	High	C	HC
6095	Jersey	1	3	-0.401	High	C	HC
5802	Jersey	3	2	-1.048	High	C	HC
6020	Jersey	2	1	0.458	Low	C	HC
5691	Jersey	3	4	2.226	Low	C	HC

*RFI* = Residual feed intake; *HC* = High concentrate; *C* = Low concentrate (control)

All cows received a low-concentrate [control (C)] and a high-concentrate (HC) diet in a crossover design with two periods (Table 9). There was approximately a 30% difference in the concentrate proportion of the dry matter (DM) basis between the high- and low-concentrate diets. In period 1, five Jersey and five Holstein cows were allocated to the high-concentrate diet, and the other four Holstein and five Jersey cows were allocated to the low-concentrate diet. Then, the animals were placed in four individual open circuit respiration chambers to measure gas exchange during the last 3 days of the trial. However, the measurements of the gases are not presented in this study. On the last day of the diet trial, the cows were transferred to a tie-a-stall area to undergo the liver biopsies.

**Table 9:** Ration composition of the experimental diet

Item	Low Concentrate	High Concentrate
Forage:Concentrate	68:32	39:61
Grass/clover silage (g/kg DM)	684	391
Barley (g/kg DM)	189	377
Rapeseed cake (g/kg DM)	25.7	51.4
Soybean meal (g/kg DM)	85.7	171
Urea (g/kg DM)	4.7	2.7
Mineral premix (g/kg DM)	9.3	5.3
Vitamin premix(g/kg DM)	2.1	1.2
Gross energy (MJ/kg DM)	18.7	19.2
DM (g/kg)	513	620
Ash (g/kg DM)	72.0	57.3
Crude protein (g/kg DM)	170	204
Crude fat (g/kg DM)	31.8	33.6
Starch (g/kg DM)	105	218
Crude fiber (g/kg DM)	179	127
NDF (g/kg DM)	335	271
iNDF (g/kg DM)	45.3	41.8

*DM* = Dry Matter; *NDF* = Neutral Detergent Fiber; *iNDF* = indigestible Neutral Detergent Fiber

After the liver biopsies, the cows were transferred and subjected to a new diet. The adaptation to the diets required 14–26 days in period 1 and 14 days in period 2. After the second diet period, another liver biopsy was performed. For the second trial, the cows were placed in a respiration chamber for 2 days at the end of the feeding trial before the transfer for the liver biopsy.

### Liver biopsy collection

Ten millilitres (ml) of Procamidor®vet (20 mg/ml) anaesthesia were injected under the skin and into the intercostal muscles at the site of the insertion of the biopsy instrument. Fifteen to 30 min after the injection, the surrounding muscle was numb, and a small incision was made through the skin in preparation for the insertion of the biopsy needle (PRO. MAG™ BIOPSY NEEDLE). Approximately 10–20 mg of liver tissue were collected from the biopsies and immersed in an RNAlater® (Sigma-Aldrich) solution for 6 days and stored at 4 °C. After 6 days, the RNAlater solution was removed, and the tissues were stored at −80 °C until further use.

### mRNA extraction and sequencing

mRNA was extracted from the liver tissue samples using the Qiazol, RNeasy® Mini Kit and MaXtract High Density for further RNA-Sequencing.

The quantity and quality of the extracted mRNA were assessed using a NanoDrop® ND-1000 spectrophotometer and Agilent 2100 Bioanalyzer machine. The quantity of the mRNA ranged from 77.95 to 1104.11 ng/μl. The quality of all mRNA samples was above 8 RIN (RNA Integrity Number). The preparation of the cDNA library and the RNA sequencing was performed by AROS Biotechnology A/S (Denmark). The cDNA originating from the RNA fragments were paired and sequenced using an Illumina HiSeq 2500 machine, and, on average, 57 million reads per sample were obtained. In detail, the fragments were paired-end sequenced, generating read pairs of 100 bp length and obtaining, on average, 28 million read pairs per sample. The RNA-Seq was performed in one run. All samples (38 samples) were pooled together using four lanes of a flow cell. The raw reads generated from the sequencing machine often were obtained in (or can be converted into) a file format called FASTQ. A read pair denotes that the sequencing was conducted from both ends of the fragment, resulting in a pair of reads, one from each end of the fragment.

### Bioinformatics and statistical analysis

**Fig. 6:**
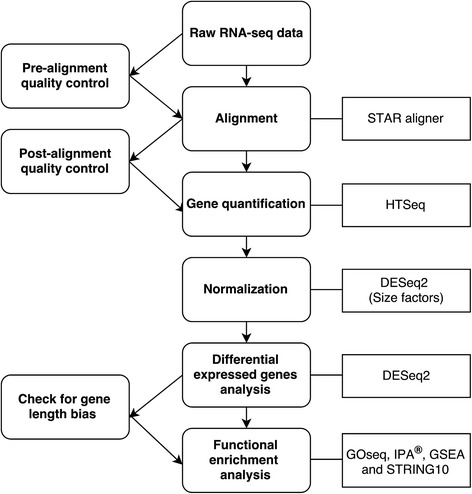
Working pipeline of the RNA-Seq analysis

The bioinformatics pipeline is shown in Fig. 6. A read quality control was conducted using FastQC version 0.11.3 [47]. Adapters were removed using cutadapt v.1.6f [48], and based on the quality control report, the reads were not further pre-processed.

Reads were aligned to the genome assembly *Bos taurus* UMD3.1 using STAR: ultrafast universal RNA-Seq aligner STAR_2.3.0 [49], providing the *Bos taurus* gene annotation file as additional information. A maximum of five mismatches was allowed, and all the other options were set as STAR default values. The reference genome and annotation file were downloaded from the Ensembl database, release 82.

A post-alignment quality control was performed on the alignment files using Qualimap version 2.0 [50]. The gene expression counts were computed using HTSeq-count [51]. This tool counts the read pairs mapping to a specific gene locus annotated in the Ensembl reference genome. Thus, we generated a matrix for each annotated gene with the corresponding raw counts. We filtered the low count genes, excluding genes with less than 1 count per million (cpm) in at least eight samples for the Holstein group and 10 samples in the Jersey group [31], where eight and 10 were the dimensions of the smallest classes in the treatment control variable in each breed.

Differentially expressed genes (DEGs) were identified using DESeq2 package version 1.12.0 [52].

The gene counts were normalized using the default normalization procedures provided by DESeq2.

The DE analyses were performed separately for each breed. All the parameters were set to default values and fitted with two different models.

1 $$ Model\ 1\kern1em  Y= Parity\  number + Diet + R F I $$

where Y is the gene expression counts, RFI is a dummy variable that represents the feed efficiency of the animals, and Parity number and Diet were codified as dummy variables included to control for potentially confounding effects. In this model, we assumed an additive effect without an interaction between diet and RFI.

2 $$ Model\ 2\kern0.75em  Y= Parity\  number + Diet + R F I + Diet: R F I $$

where Y is the gene expression counts, RFI is a dummy variable that represents the feed efficiency of the animals, and Parity number was included as a dummy variable to control for potentially confounding effects. In this model, we assumed an interaction between diet and RFI, and Diet: RFI is the interaction term (2 RFI groups × 2 treatment diets).

Differentially expressed genes were considered at a False Discovery Rate (FDR) < 5%.

A principal component analysis (PCA) was performed using the function plotPCA in the DESeq2 R package to determine the interrelations between the individual samples using the normalized counts of all the genes after filtering as the input. The PCA plot shows a strong effect of the Parity Number. Therefore, the Parity Number was included in the DE analysis to remove its confounding effect.

### Functional enrichment analysis

The functional enrichment analysis of the DEGs was performed using the GOseq version 1.24.0 package [53] in R software. Both Gene Ontology (GO) terms and the Kyoto Encyclopedia of Genes and Genomes (KEGG) pathway enrichment were used to find significant enrichment in each DEG set identified. Because of limited annotations of the bovine reference genome, orthologous human genes (Ensembl genes 82) were also used to identify the enriched GO terms and KEGG pathways.

All the significantly DEGs obtained with the DESeq2 package were used as an input for the functional enrichment analysis by QIAGEN’s Ingenuity® Pathway Analysis (IPA®, QIAGEN Redwood City, http://www.qiagen.com/ingenuity). The Entrez gene ID of a particular gene was used as input. The IPA automatically converts the *Bos taurus* Entrez ID into the corresponding human orthologous gene. We selected the top networks in each species of the network analysis in the IPA.

Finally, an additional analysis was performed using a Gene Set Enrichment Analysis (GSEA) [54, 55] from the Broad Institute that, in contrast to IPA and GOseq, considers the changes in the entire gene profile. It has been previously demonstrated that GSEA provides insight into the biology behind a set of genes in terms of how the DEGs interact with one another [56].

Furthermore, STRING 10 version 10.0 [57] was used to identify interesting associations between the significant genes identified in our study. Using the STRING database (http://string-db.org/), multiple proteins were chosen from the website interface. The DEG names were inserted as the input in the list of names, and *Bos taurus* was chosen as the organism.

## Additional files

Additional file 1: Differentially expressed gene list in Holsteins. (DOCX 23 kb)

Additional file 2: Differentially expressed gene list in Jerseys. (DOCX 17 kb)

Additional file 3: Gene Set Enrichment Analysis output. (DOCX 18 kb)

## Abbreviations

BLUP|GA: Best Linear Unbiased Prediction (approach given the Genetic Architecture)
C: Control
cDNA: Complementary DNA
CNV: Copy Number Variation
cpm: Count per million
DEG: Differentially Expressed Gene
DM: Dry Matter
eQTL: Expression Quantitative Trait Loci
FDR: False Discovery Rate
FE: Feed Efficiency
GO: Gene Ontology
GSEA: Gene Set Enrichment Analysis
GWAS: Genome Wide Association Study
HC: High Concentrate
iNDF: Indigestible Neutral Detergent Fibre
IPA®: Ingenuity® Pathway Analysis
KEGG: Kyoto Encyclopedia of Genes and Genomes
mRNA: Messenger RNA
NDF: Neutral Detergent Fiber
padj: Adjusted *p* value
PCA: Principal Component Analysis
RFI: Residual Feed Intake
RNA-Seq: RNA-Sequencing
sgBLUP: System genomic Best Linear Unbiased Prediction
SNP: Single Nucleotide Polymorphism
Tx: Treatment
