# Supplementary material for: RNA-Seq transcriptomics and pathway analyses reveal potential regulatory genes and molecular mechanisms in high- and low-residual feed intake in Nordic dairy cattle
Source: BMC Genomics. 2017 Mar 24;18:258. doi: 10.1186/s12864-017-3622-9 (PMC5366136; doi:10.1186/s12864-017-3622-9)
Supplement: Supplementary file 1 — Differentially expressed gene list in Holsteins. (DOCX 23 kb) [file 12864_2017_3622_MOESM1_ESM.docx]

| Additional file 1 Differentially expressed genes list in Holstein | | | | | |
| --- | --- | --- | --- | --- | --- |
|  | Ensembl.Gene.ID | Associated.Gene.Name | baseMean | log2FoldChange | padj |
| 1 | ENSBTAG00000000170 | Uncharacterized protein | 226.927 | -0.425 | 0.013 |
| 2 | ENSBTAG00000000654 | *ARMC4* | 58.665 | -0.591 | 0.000 |
| 3 | ENSBTAG00000001009 | *HCLS1* | 440.582 | 0.323 | 0.028 |
| 4 | ENSBTAG00000001154 | *DGAT2* | 511.454 | -0.370 | 0.029 |
| 5 | ENSBTAG00000001204 | *KIAA1462* | 225.210 | -0.416 | 0.018 |
| 6 | ENSBTAG00000002224 | *UHRF1* | 77.588 | -0.496 | 0.000 |
| 7 | ENSBTAG00000002526 | *BDH2* | 1382.085 | -0.579 | 0.000 |
| 8 | ENSBTAG00000002705 | *REC8* | 304.679 | -0.365 | 0.002 |
| 9 | ENSBTAG00000003696 | *CCDC64* | 45.091 | 0.445 | 0.027 |
| 10 | ENSBTAG00000003718 | *HACL1* | 6329.552 | 0.315 | 0.039 |
| 11 | ENSBTAG00000004076 | *OXER1* | 223.678 | -0.435 | 0.010 |
| 12 | ENSBTAG00000004558 | *C15orf48* | 89.057 | 0.505 | 0.003 |
| 13 | ENSBTAG00000004908 | *CHRNE* | 246.499 | -0.749 | 0.000 |
| 14 | ENSBTAG00000005287 | *CYP7A1* | 4126.209 | 0.456 | 0.013 |
| 15 | ENSBTAG00000005629 | *AIM1L* | 913.588 | -0.298 | 0.003 |
| 16 | ENSBTAG00000006452 | *CD3D* | 77.747 | 0.407 | 0.037 |
| 17 | ENSBTAG00000006599 | *INHBE* | 605.947 | -0.424 | 0.044 |
| 18 | ENSBTAG00000006675 | *PCSK6* | 3039.353 | -0.187 | 0.028 |
| 19 | ENSBTAG00000006934 | *CYP11A1* | 649.881 | 0.486 | 0.005 |
| 20 | ENSBTAG00000006978 | *HSD17B4* | 13797.370 | 0.301 | 0.027 |
| 21 | ENSBTAG00000006999 | *RYR1* | 148.344 | 0.517 | 0.002 |
| 22 | ENSBTAG00000007554 | *IFI6* | 136.813 | 0.376 | 0.049 |
| 23 | ENSBTAG00000007828 | *SLA* | 118.256 | 0.323 | 0.039 |
| 24 | ENSBTAG00000007895 | *SLC20A1* | 880.080 | -0.563 | 0.000 |
| 25 | ENSBTAG00000008160 | *MBOAT2* | 440.595 | 0.344 | 0.044 |
| 26 | ENSBTAG00000008424 | *ABR* | 459.604 | 0.329 | 0.032 |
| 27 | ENSBTAG00000008913 | *TMEM98* | 333.139 | -0.518 | 0.001 |
| 28 | ENSBTAG00000009085 | *SLC35A5* | 1691.358 | 0.278 | 0.007 |
| 29 | ENSBTAG00000009137 | *NKG7* | 215.444 | 0.381 | 0.028 |
| 30 | ENSBTAG00000009263 | *MFSD1* | 2661.739 | 0.240 | 0.013 |
| 31 | ENSBTAG00000010463 | Uncharacterized protein | 394.139 | 0.384 | 0.002 |
| 32 | ENSBTAG00000010564 | *ELOVL6* | 994.737 | 0.439 | 0.027 |
| 33 | ENSBTAG00000011771 | *FICD* | 107.908 | -0.360 | 0.037 |
| 34 | ENSBTAG00000011832 | *ALDH18A1* | 404.909 | 0.313 | 0.026 |
| 35 | ENSBTAG00000012007 | *SOCS2* | 835.321 | 0.422 | 0.044 |
| 36 | ENSBTAG00000012995 | *CCDC109B* | 52.933 | 0.424 | 0.037 |
| 37 | ENSBTAG00000013596 | *NR1H4* | 1215.489 | 0.241 | 0.012 |
| 38 | ENSBTAG00000014064 | *FGFR2* | 1554.530 | -0.402 | 0.001 |
| 39 | ENSBTAG00000014791 | *CTH* | 224.753 | -0.535 | 0.000 |
| 40 | ENSBTAG00000015313 | *CEACAM19* | 51.603 | -0.944 | 0.000 |
| 41 | ENSBTAG00000015419 | *ARHGEF37* | 204.979 | 0.472 | 0.002 |
| 42 | ENSBTAG00000016542 | *LAMB3* | 1783.587 | 0.425 | 0.025 |
| 43 | ENSBTAG00000017567 | *ACACA* | 844.772 | 0.404 | 0.005 |
| 44 | ENSBTAG00000018116 | *MTFP1* | 88.162 | -0.387 | 0.026 |
| 45 | ENSBTAG00000018548 | *INTS7* | 6522.273 | 0.238 | 0.002 |
| 46 | ENSBTAG00000018604 | *SEMA4G* | 4847.004 | -0.161 | 0.049 |
| 47 | ENSBTAG00000018723 | *SLC25A34* | 96.514 | -0.442 | 0.029 |
| 48 | ENSBTAG00000019585 | *MYOM1* | 962.386 | 0.453 | 0.025 |
| 49 | ENSBTAG00000020116 | *JSP.1* | 2041.873 | 0.324 | 0.017 |
| 50 | ENSBTAG00000020371 | *ACOT8* | 312.398 | 0.418 | 0.013 |
| 51 | ENSBTAG00000020375 | Uncharacterized protein | 5129.183 | 0.422 | 0.031 |
| 52 | ENSBTAG00000020499 | Uncharacterized protein | 68.214 | 0.572 | 0.000 |
| 53 | ENSBTAG00000020755 | *SELP* | 478.821 | -0.392 | 0.015 |
| 54 | ENSBTAG00000021746 | *ANXA5* | 333.856 | -0.384 | 0.039 |
| 55 | ENSBTAG00000023851 | *FAM102A* | 229.007 | -0.510 | 0.001 |
| 56 | ENSBTAG00000023929 | *FOSL2* | 189.622 | 0.424 | 0.028 |
| 57 | ENSBTAG00000024044 | *CDKL4* | 82.117 | 0.515 | 0.002 |
| 58 | ENSBTAG00000025258 | Uncharacterized protein | 102.690 | 0.544 | 0.002 |
| 59 | ENSBTAG00000025898 | *TBC1D8* | 442.302 | 0.272 | 0.048 |
| 60 | ENSBTAG00000026779 | *LYZ* | 516.844 | 0.644 | 0.000 |
| 61 | ENSBTAG00000030966 | *TAF6* | 419.594 | -0.256 | 0.010 |
| 62 | ENSBTAG00000035998 | *CKB* | 332.043 | 0.385 | 0.049 |
| 63 | ENSBTAG00000037913 | Uncharacterized protein | 436.527 | 0.212 | 0.043 |
| 64 | ENSBTAG00000037917 | *SLC17A1* | 2786.405 | 0.438 | 0.017 |
| 65 | ENSBTAG00000038496 | *CR2* | 1355.757 | -0.542 | 0.000 |
| 66 | ENSBTAG00000038962 | *SLC6A11* | 2637.353 | -0.370 | 0.010 |
| 67 | ENSBTAG00000039731 | *RND3* | 1761.210 | -0.254 | 0.028 |
| 68 | ENSBTAG00000046076 | Uncharacterized protein | 124.754 | -0.420 | 0.048 |
| 69 | ENSBTAG00000046730 | Uncharacterized protein | 139.827 | 0.365 | 0.049 |
| 70 | ENSBTAG00000047529 | Uncharacterized protein | 110.838 | -0.535 | 0.002 |
| +v e log2 fold change = upregulated in low feed efficiency (high RFI) group | | | | | |
| -ve log2 fold change = downregulated in low feed efficiency (high RFI) group | | | | | |
