# Supplementary material for: RNA-Seq transcriptomics and pathway analyses reveal potential regulatory genes and molecular mechanisms in high- and low-residual feed intake in Nordic dairy cattle
Source: BMC Genomics. 2017 Mar 24;18:258. doi: 10.1186/s12864-017-3622-9 (PMC5366136; doi:10.1186/s12864-017-3622-9)
Supplement: Supplementary file 2 — Differentially expressed gene list in Jerseys. (DOCX 17 kb) [file 12864_2017_3622_MOESM2_ESM.docx]

| Additional file 2 Differentially expressed genes list in Jersey | | | | | |
| --- | --- | --- | --- | --- | --- |
|  | Ensembl.Gene.ID | Associated.Gene.Name | baseMean | log2FoldChange | padj |
| 1 | ENSBTAG00000006525 | *FDXR* | 125.970 | -0.645 | 0.000 |
| 2 | ENSBTAG00000008066 | *PKDREJ* | 76.750 | 0.562 | 0.000 |
| 3 | ENSBTAG00000013689 | *MCTP2* | 148.262 | 0.529 | 0.000 |
| 4 | ENSBTAG00000027727 | Uncharacterized protein | 284.200 | 0.480 | 0.000 |
| 5 | ENSBTAG00000038487 | *ZNF613* | 155.398 | -0.390 | 0.026 |
| 6 | ENSBTAG00000046257 | *GIMAP4* | 650.179 | -0.387 | 0.002 |
| 7 | ENSBTAG00000005182 | *BOLA-A* | 434.954 | -0.387 | 0.001 |
| 8 | ENSBTAG00000014402 | *GIMAP8* | 713.049 | -0.382 | 0.009 |
| 9 | ENSBTAG00000045727 | Uncharacterized protein | 921.104 | 0.381 | 0.032 |
| 10 | ENSBTAG00000019026 | *EXTL2* | 34.590 | 0.378 | 0.037 |
| 11 | ENSBTAG00000037440 | *ZNF197* | 281.101 | 0.358 | 0.016 |
| 12 | ENSBTAG00000021751 | *RASEF* | 36.023 | -0.350 | 0.011 |
| 13 | ENSBTAG00000027205 | *PGBD5* | 30.062 | -0.340 | 0.026 |
| 14 | ENSBTAG00000031737 | *TMEM102* | 26.530 | 0.339 | 0.037 |
| 15 | ENSBTAG00000009087 | *GNG10* | 1516.440 | -0.325 | 0.026 |
| 16 | ENSBTAG00000040323 | Uncharacterized protein | 1003.599 | -0.321 | 0.026 |
| 17 | ENSBTAG00000014161 | *ARMC10* | 258.840 | -0.298 | 0.026 |
| 18 | ENSBTAG00000013106 | *C19orf81* | 26.345 | 0.295 | 0.026 |
| 19 | ENSBTAG00000047379 | *CYP3A4* | 2422.366 | 0.287 | 0.043 |
| +v e log2 fold change = upregulated in low feed efficiency (high RFI) group | | | | | |
| -ve log2 fold change = downregulated in low feed efficiency (high RFI) group | | | | | |
