# Supplementary material for: RNA-Seq transcriptomics and pathway analyses reveal potential regulatory genes and molecular mechanisms in high- and low-residual feed intake in Nordic dairy cattle
Source: BMC Genomics. 2017 Mar 24;18:258. doi: 10.1186/s12864-017-3622-9 (PMC5366136; doi:10.1186/s12864-017-3622-9)
Supplement: Supplementary file 3 — Gene Set Enrichment Analysis output. (DOCX 18 kb) [file 12864_2017_3622_MOESM3_ESM.docx]

Additional file 3 Gene Set Enrichment Analysis (GSEA) output

| Downregulated KEGG pathways for FDR q-value <0.05 from the output of GSEA in Holstein | | |
| --- | --- | --- |
|  | Name | FDR q-value |
| 1 | Primary immunodeficiency | ~0 |
| 2 | Natural killer cell mediated cytotoxicity | ~0 |
| 3 | T cell receptor signaling pathway | ~0 |
| 4 | Leukocyte transendothelial migration | 0.002 |
| 5 | Chemokine signaling pathway | 0.002 |
| 6 | FC gamma R mediated phagocytosis | 0.008 |
| 7 | Propanoate metabolism | 0.009 |
| 8 | Rig I like receptor signaling pathway | 0.013 |
| 8 | Cell adhesion molecules cams | 0.012 |
| 9 | Calcium signaling pathway | 0.012 |
| 10 | B cell receptor signaling pathway | 0.021 |
| 11 | Nod like receptor signaling pathway | 0.028 |
| 12 | Viral myocarditis | 0.031 |
| 13 | FC epsilon RI signaling pathway | 0.038 |
| 14 | Leishmania infection | 0.043 |

| Downregulated KEGG pathways for FDR q-value <0.05 from the output of GSEA in Jersey | | |
| --- | --- | --- |
|  | Pathways name | FDR q-value |
| 1 | Leukocyte transendothelial migration | 0.006 |
| 2 | Primary immunodeficiency | 0.010 |
| 3 | Cytosolic DNA sensing pathway | 0.013 |
| 4 | Leishmania infection | 0.015 |
| 5 | Hematopoietic cell lineage | 0.044 |

| Upregulated KEGG pathways for FDR q-value <0.05 from the output of GSEA in Jersey | | |
| --- | --- | --- |
|  | Pathways name | FDR q-value |
| 1 | Retinol metabolism | 0.002 |
| 2 | Metabolism of xenobiotics by cytochrome P450 | 0.003 |
| 3 | Ether lipid metabolism | 0.009 |
| 4 | Starch and sucrose metabolism | 0.012 |
| 5 | Steroid hormone biosynthesis | 0.013 |
| 6 | Sphingolipid metabolism | 0.015 |
| 7 | Arachidonic acid metabolism | 0.023 |
| 8 | Glycolysis gluconeogenesis | 0.025 |
| 9 | Drug metabolism cytochrome P450 | 0.029 |
| 10 | Pentose phosphate pathway | 0.029 |
